# Normative values and factors affecting Pediatric Reach Tests in Saudi children aged 6–11 years in the eastern province: cross-sectional study

**DOI:** 10.3389/fped.2023.1240659

**Published:** 2024-01-04

**Authors:** Reem S. Alotaibi, Maha F. Algabbani, Afaf A. M. Shaheen, Alaa M. Albishi, Muneera M. Almurdi

**Affiliations:** ^1^ Department of Health Rehabilitation Sciences, College of Applied Medical Sciences, King Saud University, Riyadh, Saudi Arabia; ^2^ Basic Science Department, Faculty of Physical Therapy, Cairo University, Cairo, Egypt

**Keywords:** Pediatric Reach Tests, normative values, postural control, dynamic balance, typically developing Saudi children, Saudi Arabia

## Abstract

**Background:**

The Pediatric Reach Tests (PRTs) assess balance while standing—the Functional Reach Test (FRT) and Lateral Reach Test (LRT)—and in a sitting position—the Modified Functional Reach Test (MFRT) and Modified Lateral Reach Test (MLRT). Normative values have not been fully evaluated in Saudi children. The objectives are; to estimate the normative values for PRTs; investigate the correlation between the PRTs and demographic/anthropometric characteristics; and develop predictive equations for the PRTs.

**Methods:**

In this cross-sectional study, 251 children aged 6–11 were recruited. The PRTs were measured and correlated with demographic/anthropometric variables. A stepwise regression was conducted to develop the predictive equations for the PRT scores.

**Results:**

The mean and standard deviations (in cm) of the PRT scores were as follows: FRT = 20.02 ± 4.31; LRT = 13.42 ± 3.38; MFRT = 21.49 ± 4.70, and MLRT = 14.64 ± 3.66. Several significant correlations were found. Moderate correlations existed between the PRT scores and age, height, upper extremity length, lower extremity length, and foot length; there was a weak correlation with body mass index. Weight was moderately correlated with FRT and MFRT and weakly correlated with LRT and MLRT. The correlation between the base of support and LRT was moderate and was weak with FRT, MFRT, and MLRT. A weak correlation was found between sex and LRT. Age and height were the most predictive of PRT scores.

**Conclusion:**

This study provided PRT normative values that can be used as a clinical reference for evaluating balance in typically developing children.

## 1 Introduction

Balance is a fundamental component of human movements; it is the process by which postural stability is maintained over the base of support (BOS) (1). Maintaining balance is essential for children during movements (1). Many pediatric neurological disorders have been associated with balance impairments, such as cerebral palsy (CP), Down's syndrome (DS), traumatic brain injury (TBI), spinal cord injury (SCI), and hearing impairments (2–5).

Clinically, balance can be evaluated through many balance assessment approaches for children with or without disabilities, such as the Pediatric Balance Scale (PBS), modified TUG (mTUG) test, and Pediatric Reach Tests (PRTs) (6–10).

One of the several components of balance that the reaching test addresses is anticipatory balance. Anticipatory balance refers to the ability to maintain stability and prevent falls by making adjustments in preparation for postural changes. It involves the coordination of sensory information, motor responses, and cognitive processes to effectively adapt to changes in the environment (11).

The PRTs are simple, valid, and time-efficient clinical tools that measure how far an individual can reach (forward or laterally) without losing balance while standing or sitting (9).

The PRTs consist of four reaching tests. There are two from the standing position: the Functional Reach Test (FRT) and the Lateral Reach Test (LRT). There are also two from a sitting position: the Modified Functional Reach Test (MFRT) and the Modified Lateral Reach Test (MLRT) (9).

Normative values for reaching tests are useful for both clinicians and researchers; they provide a basis for comparison and help in the diagnosis of potential balance deficits (12). Studies have established the normative values for different reach tests: the FRT and LRT for Saudi children in two different regions (13, 14); the FRT, LRT, MFRT, and MLRT for Indian children (15, 16); and the FRT and LRT for Turkish children (17). Previous studies revealed that there is evidence of differences in normative reaching test scores among different populations (14, 15, 17) that could be a result of environmental factors, nutrition, or variation in growth or puberty. In Saudi Arabia (SA), the scores on the reaching test may vary from region to region because of factors such as these. Normative values for MRT and MLRT have not been studied in Saudi Arabia.

Previous studies have reported that age, sex, height, weight, body mass index (BMI), upper extremity (UE) length, lower extremity (LE) length, foot length, and BOS could influence PRT scores (13–17).These factors were found to be different between various populations (14, 16, 18). Therefore, local normative values can best reflect the ethnic characteristics of a specific population.

Thus, our research aims were to expand the existing research by establishing normative values for the PRTs among Saudi typical developing (TD) children aged 6–11 years old in the Eastern Province, SA; to evaluate the association between PRT scores and demographic variables (age and sex) and anthropometric measures (height, weight, BMI, UE, LE, FL, and BOS); and to develop predictive equations for estimated PRT scores.

## 2 Materials and methods

### 2.1 Design and setting

This was an observational cross-sectional study. A convenience sample was recruited from five governmental and private schools randomly selected from three cities (Khobar, Dhahran, and Dammam) in the Eastern Province. The study was conducted in the participants’ schools. Data were collected from January to March 2022.

### 2.2 Participants

School-age children were divided into six groups according to their age in 1-year increments. Each group included children of the stated age, whether they had just had that birthday or were closer to their next one; for example, the 11-year-olds group contained recent 11-year-olds and those even one day before their 12th birthday. Each group was divided into two sub-groups according to sex.

All the participants were TD Saudi children aged 6–11 years and represented both sexes. Children with a history of neurological or orthopedic conditions; balance impairment; visual disorders; middle-ear infection; or hip, knee, or ankle injury within the past six months were excluded (13–17).

### 2.3 Sample size

The sample size was calculated using a “rule of thumb” method, according to the following equation: *N *≥ 104 + *M*, where *N* = sample size and *M* = the number of independent variables (sex, age, height, weight, BMI, UE and LE length, foot length, and BOS). Based on this equation, the minimum sample size required was estimated to be 113 (19). To study both sexes, at least 226 children were needed, which is an appropriate sample size for conducting the regression analysis (20). According to G-Power Calculation (version 3.1.9.4), the sample size was also sufficient for MANOVA to find a medium effect size of 0.5 (based on Cohen's d), a significance level of 0.05, and a power level of 0.95. Therefore, at least 38 children of both sexes in each age group were required.

### 2.4 Ethical consideration and consent

The study was approved by the Institutional Review Board (IRB) at King Saud University (KSU) (No. E-21-5745) and by the Ministry of Education (No. 4300170391). A written consent form was signed by the children's legal guardians before participation. Moreover, assent was obtained from the children as appropriate.

### 2.5 Procedures

Invitation letters and consent forms were sent to the legal guardians of the children through the school administration. The study was conducted in a classroom with one empty wall on which a leveled meter stick was attached at the child's shoulder level. The participants were asked to wear light clothes with exposed shoulders and to remove their shoes and socks. Data collection was done by the same physical therapist (the principal investigator).

#### 2.5.1 Demographic and anthropometric measurement

Demographic data sheets and general health questions were collected from the participants' legal guardians. A tape measure was used to determine the accurate height; UE, LE, and foot length; and BOS in centimeters (17). Each participant's weight in kilograms (kg) was measured using a portable digital weight scale (TANITA HD 378). The BMI was calculated based on height and weight (21), using the formula:

$$\mathrm{BMI}\;=\;\frac{\mathrm{Weight}\;(\mathrm{kg})}{\mathrm{Height}\;\mathrm{squared}\;(m^{2})}$$

UE length was measured for the dominant arm from the tip of the acromion process to the tip of the middle finger. LE length was measured from the anterior superior iliac spine to the tip of the medial malleolus. Determining the BOS involved measuring the distance between the two acromion processes of the shoulders (9). The foot length of the dominant side was measured as the distance from the back of the child's heel to the tip of the big toe (17).

#### 2.5.2 Procedure of performing PRTs

The administration of the PRTs took 15–20 min, with 60 s of rest between the tests (9), based on the procedure described in the literature (9, 14, 16). Lateral reaching was performed on the child's right side (9, 15–17). The child's feet were traced on a piece of paper taped to the floor, within the BOS, to ensure the foot position.

The child was instructed to reach with his/her fisted right hand as far as he/she could. The other arm was held in a neutral position next to the body. The therapist demonstrated the test to the child. The child had one practice trial and three recorded trials with 10–15 s of rest between them; the mean of three successful trials was calculated. The test trial was repeated if the child took a step, touched a wall or the therapist, or lifted his/her heels. The starting and ending positions were held for three seconds each, and the level of the third metacarpal head along with the meter stick was recorded to the nearest centimeter. The difference between the starting and ending positions was also recorded.

**Figure 1:**
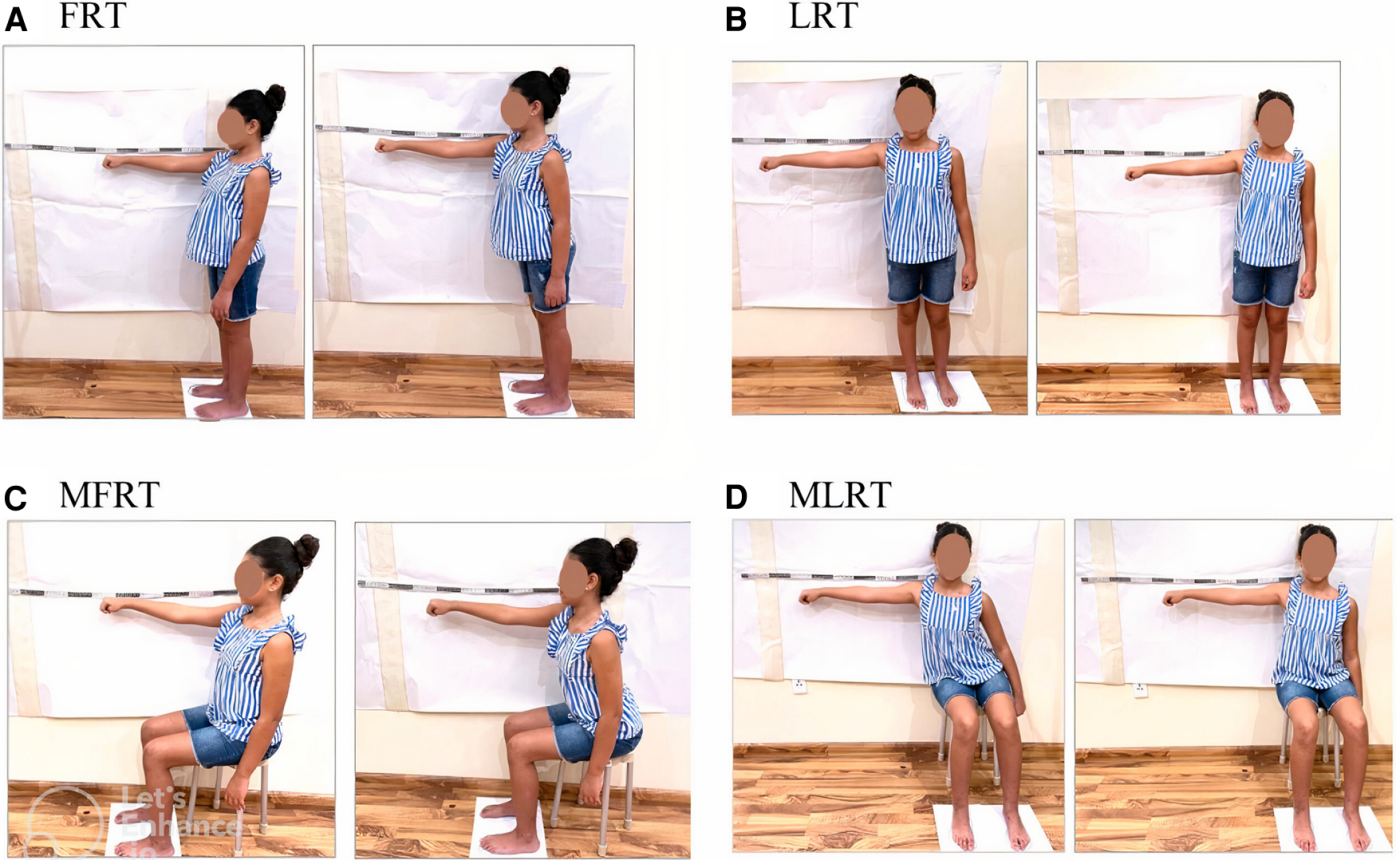
PRTs: (**A**) functional reach test (FRT), (**B**) lateral reach test (LRT), (**C**) modified functional reach test (MFRT), and (**D**) modified lateral reach test (MLRT).

During the FRT, the child stood with his/her right side beside the wall, shoulder at 90° of forward flexion, elbow extended, wrist in the neutral position, and hand closed in a fist; he/she was then asked to reach forward. During the LRT, the child stood against the wall with his/her shoulder abducted 90° and asked to reach laterally. During the MFRT, the child sat on a backless chair without armrests, back straight, hips and knees flexed 90°, feet flat on the floor, and feet separated by pelvis width; they were then asked to reach forward. During the MLRT, the child sat with his/her back toward the wall, without touching it, with his/her shoulder abducted 90° and asked to reach laterally (see Figure 1).

### 2.6 Statistical analysis

Statistical analyses were conducted using IBM SPSS Statistics for Windows, Version 28.0 (Armonk, NY: IBM Corp). The Shapiro–Wilk test was used to assess the normality of continuous variables. Data were presented as means and standard deviations (SD) for normally distributed data or as quartiles [1st, 2nd (median), and 3rd] for skewed data. Frequencies and percentages were used for categorical data. To compare the sexes, an independent sample *t*-test was used. A two-way analysis of variance (ANOVA) was calculated to assess the main effect of sex, age, and sex × age interaction on the PRT scores. Partial eta-squared values (*η*^2^) of .01, .06, and .14 represented small, medium, and large effect sizes, respectively (22). Bonferroni Post hoc analysis was utilized to identify the differences in every pair-wise condition. The correlations between the PRT scores and the independent variables were tested using Pearson's (*r*) and Spearman's (rho) correlations according to the data normality. Eta (*η*) was used to assess the correlation with sex. Correlation coefficients were interpreted as follows: no correlation,  < .1; weak, .1–.3; moderate, .4–.6; strong, .7–.8; and a perfect correlation = 1 (23). Variables that showed significant association were included in the forward stepwise linear regression analysis to determine the predicted variables for the PRT scores. At each step, variables were added based on *p*-values and the absence of multicollinearity. Multicollinearity was checked using the variance inflation factor (VIF) with a cut-off point of 5 (24). With a *p*-value < 0.05, all results were considered statistically significant.

## 3 Results

A total of 760 consent forms were distributed to the participants, and 302 forms were received. Fifty-one participants were excluded for various reasons [musculoskeletal injuries (*n* = 8), balance impairment (*n* = 2), middle-ear infection (*n* = 1), and declined to participate (*n* = 40)]. In total, 251 participants were included in the study (135 boys and 116 girls).

### 3.1 Participants' characteristics and PRTs scores

The characteristics of the participants and PRT scores are presented in Table 1. All the data were homogenous and normally distributed, except for foot length and BOS. The means and standard deviations of the PRT scores were as follows: FRT, 20.02 ± 4.31 cm; LRT, 13.42 ± 3.38; MFRT, 21.49 ± 4.70 cm; and MLRT 14.64 ± 3.66 cm. Generally, there were no significant differences in age or anthropometric measures between boys and girls (*p* > .05). However, boys in the 7-year-olds group were significantly heavier than girls in the same age group (27.81 kg vs. 24.25 kg, *p* < .05), and girls in the 11-year-olds group were significantly taller than boys in the same age group (153.61 cm vs. 144.30 cm, *p *< .01).

**Table 1:** Participants’ demographic and anthropometric characteristics and normative values of PRTs across age and sex.

Age (yrs)	Sex	*N*	Height (cm)	Weight (kg)	BMI	UE length (cm)	LE length (cm)	Foot length (cm)	BOS (cm)	FRT (cm)	LRT (cm)	MRT (cm)	MLRT (cm)
6	B	20	120.50 ± 5.75	22.75 ± 3.31	15.59 ± 1.28	52.80 ± 2.78	62.40 ± 3.19	19.00 (19, 20)	31.50 (31, 33)	15.26 ± 3.59	12.11 ± 2.34*	16.80 ± 4.80	12.04 ± 2.31
	G	19	119.53 ± 4.96	22.68 ± 4.64	15.77 ± 2.03	52.68 ± 2.71	62.58 ± 3.31	19.00 (18, 20)	31.00 (29, 32)	16.11 ± 3.51	10.05 ± 1.84	17.93 ± 2.81	12.11 ± 2.80
7	B	26	127.50 ± 4.75	27.81 ± 5.29*	16.97 ± 2.83	56.65 ± 2.06	67.04 ± 3.18	20.00 (19, 21)	33.00 (32, 43.25)	18.77 ± 3.44	12.36 ± 3.16*	20.15 ± 3.83	12.49 ± 3.72
	G	20	124.55 ± 6.30	24.25 ± 4.75	15.50 ± 2.18	54.70 ± 3.39	66.10 ± 4.33	19.00 (18, 20)	31.00 (30, 33)	16.86 ± 3.53	10.23 ± 2.07	17.93 ± 3.30	12.05 ± 1.53
8	B	20	129.90 ± 8.39	26.90 ± 5.26	15.77 ± 1.73	57.45 ± 3.94	67.60 ± 5.91	20.00 (19.25, 21)	33.00 (31.25, 35.75)	19.87 ± 3.67	13.27 ± 2.93	21.71 ± 4.35	14.70 ± 3.06
	G	20	131.70 ± 5.58	29.30 ± 5.66	16.81 ± 2.43	58.05 ± 2.56	69.70 ± 4.13	21.00 (20.25, 22)	33.00 (33, 34.75)	20.84 ± 3.75	12.29 ± 2.53	22.27 ± 4.51	15.56 ± 3.13
9	B	26	136.38 ± 6.11	36.19 ± 8.64	19.33 ± 3.79	61.27 ± 3.09	73.58 ± 4.37	22.00 (21, 23)	35.00 (33, 37)	21.53 ± 3.70	14.84 ± 3.23	22.99 ± 5.04	16.09 ± 2.94
	G	19	137.84 ± 4.61	33.63 ± 7.55	17.62 ± 3.47	61.84 ± 2.67	75.21 ± 3.29	21.00 (21, 22)	34.00 (32, 35)	21.77 ± 3.46	13.74 ± 2.61	23.53 ± 3.78	15.28 ± 3.65
10	B	23	141.39 ± 5.91	36.83 ± 7.73	18.33 ± 3.30	62.65 ± 3.90	75.39 ± 3.69	22.00 (22, 23)	36.00 (34, 37)	21.70 ± 3.58	15.49 ± 2.48	23.67 ± 3.48	16.82 ± 3.06
	G	20	143.30 ± 8.60	36.80 ± 7.42	17.75 ± 2.09	64.15 ± 4.0	77.95 ± 5.99	22.00 (21, 23)	36.50 (35, 38)	21.57 ± 2.39	14.26 ± 2.83	22.94 ± 4.27	15.95 ± 3.81
11	B	20	144.30 ± 6.97	40.20 ± 8.08	19.28 ± 3.00	64.75 ± 4.52	79.60 ± 5.23	24.00 (22, 25)	37.50 (35, 39.75)	23.57 ± 3.51	16.96 ± 2.86	23.42 ± 4.52	16.74 ± 3.95
	G	18	153.61 ± 3.42*	42.44 ± 4.44	17.97 ± 2.29	69.94 ± 2.34	84.67 ± 2.57	23.00 (23, 24)	39.00 (36.75, 39.25)	22.16 ± 4.66	15.18 ± 3.75	24.26 ± 4.41	15.70 ± 3.99
Total boys		135	133.39 ± 10.11	31.91 ± 9.01	17.61 ± 3.19	59.31 ± 5.15	70.98 ± 7.03	21 (20, 23)	34 (32, 46)	20.15 ± 4.34	14.15 ± 3.31*	21.49 ± 4.67	14.81 ± 3.71
Total girls		116	134.88 ± 12.68	31.39 ± 8.97	16.89 ± 2.59	60.11 ± 6.49	72.56 ± 8.40	21 (19.25, 22)	34 (31, 36)	19.86 ± 4.28	12.56 ± 3.27	21.48 ± 4.55	14.43 ± 3.60
Total		251	134 ± 11.37	31.67 ± 8.98	17.28 ± 2.95	59.68 ± 5.81	71.71 ± 7.72	21 (20, 23)	34 (32, 36)	20.02 ± 4.31	13.42 ± 3.38	21.49 ± 4.70	14.64 ± 3.66

N, number of participants; B, boys; G, girls; yrs, years; cm, centimeters; Kg, kilograms; BMI, body mass index; UE, upper extremity; LE, lower extremity; BOS, base of support; FRT, forward reach test; LRT, lateral reach test; MFRT, modified forward reach test; MLRT, modified lateral reach test; SD, standard deviation; Q, quartile.

Data are presented as mean ± SD, except Foot Length and BOS are presented as median and (1st and 3rd Q). Comparison between boys and girls in the same group and for the total using independent sample *t*-test (except foot length and BOS, Mann–Whitney test was used).

* Significant at .05.

### 3.2 The PRT scores by age and sex

A two-way ANOVA yielded a significant main effect of age (*p* < .001) in all the PRTs as follows: FRT, *F*_(5,239) _= 23.22, *p* < .001; LRT, *F*_(5,239) _= 22.67, *p* < .001; MFRT, *F*_(5,239) _= 16.25, *p* < .001; and MLRT, *F*_(5,239) _= 15.16, *p* < .001. Generally, the PRT scores increased with age (see Table 2).

**Table 2:** Two-way ANOVA testing the effect of age and sex in PRTs.

Source of variation	Df	FRT			LRT			MFRT			MLRT		
		*F*	*P*	*η* ^2^	*F*	*P*	*η* ^2^	*F*	*P*	*η* ^2^	*F*	*P*	*η* ^2^
Age	5	23.22	<.001	.33	22.67	<.001	.32	16.25	<.001	.25	15.16	<.001	.24
Sex	1	0.25	.62	.001	21.64	<.001	.08	0.003	.96	.000	0.82	.37	.003
Interaction effect (Age × Sex)	5	1.14	.34	.23	0.52	.76	.011	0.83	.53	.02	0.50	.78	.01
Error		239											
Total		251											
Correct total		250											

FRT, forward reach test; LRT, lateral reach test; MFRT, modified forward reach test; MLRT, modified lateral reach test; Df, degrees of freedom; F, *F*-test; *η*_p_^2^, effect size.

*P* = significance level.

Table 3 shows the Post hoc analysis revealed that there were significant differences in the FRT, MFRT, and MLRT scores between children aged 6 and 7 years and older. In addition, there were significant differences in LRT scores between children aged 6–8 years and older (*p* < .001). Moreover, the 8-year-old children's FRT scores significantly differed from those of the 11-year-old children, and their LRT scores significantly differed from those of the 10 and 11-year-old children. There were no significant differences in the four PRT values between children aged 6–7 years and children aged 9–11 years (*p* > .05).

**Table 3:** Bonferroni post hoc analysis for every pair-wise age group.

	Age (years)	7	8	9	10	11
FRT	**6**	.06	<.001*	<.001*	<.001*	<.001*
	**7**		.03*	<.001*	<.001*	<.001*
	**8**			1.00	1.00	.03*
	**9**				1.00	1.00
	**10**					1.00
LFRT	**6**	1.00	.27	<.001*	<.001*	<.001*
	**7**		.20	<.001*	<.001*	<.001*
	**8**			.16	.01*	<.001*
	**9**				1.00	.09
	**10**					.91
MRT	**6**	1.00	<.001*	<.001*	<.001*	<.001*
	**7**		.03*	<.001*	<.001*	<.001*
	**8**			1.00	.91	1.00
	**9**				1.00	1.00
	**10**					1.00
LMRT	**6**	1.00	<.001*	<.001*	<.001*	<.001*
	**7**		<.001*	<.001*	<.001*	<.001*
	**8**			1.00	1.00	1.00
	**9**				1.00	1.00
	**10**					1.00

FRT, forward reach test; LRT, lateral reach test; MFRT, modified forward reach test; MLRT, modified lateral reach test.

* Significant at .05.

There was no main effect of sex on the PRT scores (*p*-values ranged from .37 to .96), except LRT [*F*_(1,239) _= 21.64, *p* < .001], in which boys performed significantly better than girls. However, the effect size of sex on LRT was very small (*η*^2 ^= .08). Furthermore, there were no significant age × sex interactions (*p*-values ranged from .23 to .78).

### 3.3 Factors affecting the performance of PRTs

Table 4 shows the correlations of the PRT scores with the demographic and anthropometric variables. All the correlations were positive, and except for those involving sex, all were significant at the .01 level. All the PRT scores had moderate correlations with age, height, UE length, LE length, and foot length, and weak correlations with BMI. There were moderate correlations between weight and both FRT and MFRT and weak correlations with both LRT and MLRT. There was a moderate correlation between BOS and LRT, and weak correlations with FRT, MFRT, and MLRT. In addition, there was a significant but weak correlation between sex and LRT and non-significant correlations of sex with FRT, MFRT, and MLRT.

**Table 4:** Correlations between the PRT and independent variables.

Variables	Correlation test	FRT	LRT	MFRT	MLRT
Sex	Eta test	.03	.24**	.01	.05
Age	Pearson (*r*)	.59**	.53**	.48**	.46**
Height		.57**	.46**	.51**	.47**
Weight		.44**	.38**	.41**	.31**
BMI		.19**	.19**	.20**	.08**
UE length		.54**	.41**	.48**	.41**
LE length		.55**	.47**	.47**	.44**
Foot length	Spearman (rho)	.49**	.53**	.45**	.44**
BOS		.38**	.42**	.34**	.29**

FRT, functional reach test; LRT, lateral reach test; MFRT, modified functional reach test; MLRT, modified lateral reach test; BMI, body mass index; UE, upper extremity; LE, lower extremity; BOS, base of support.

** Indicates correlations significant at the .01 level (2-tailed).

### 3.4 Predicting factors of PRTs

Height and age were the most predictive variables for FRT, MFRT, and MLRT (*R* = .59, .50, and .49 respectively). Age was the predictive variable for LRT (*R* = .52) (Table 5). The following are the predictive equations for the PRTs:

• **FRT** (cm) = −3.22 + [0.12 × height (cm)] + [0.75 × age (years)]; *r*^2 ^= .34.
• **LRT** (cm) = 3.83 + [1.10 × age (years)]; *r*^2 ^= .27.
• **MFRT** (cm) = −3.89 [0.17 × height (cm)] + [0.35 × age (years)]; *r*^2 ^= .25.
• **MLRT** (cm) = −1.94 + [0.09 × height (cm)] + [0.51 × age (years)]; *r*^2 ^= .24.

**Table 5:** Linear regression analysis for predicting PRT scores.

Test	Model	Independent variables	*R*	*R* ^2^	Unstandardized coefficient		Standardized coefficient β	*p*
					B	SE		
FRT	1	(Constant)	.57	.32	−8.71	2.66	0.57	.001
		Height			0.21	0.02		<.001
	2	(Constant)	.59	.34	−3.22	3.23	0.33	.32
		Height			0.12	0.04	0.28	<.001
		Age			0.75	0.26		.004
LRT	1	(Constant)	.52	.27	3.83	1.02	0.52	<.001
		Age			1.10	0.11		<.001
MFRT	1	(Constant)	.49	.24	−6.45	3.14	0.49	.04
		Height			0.21	0.02		<.001
	2	(Constant)	.50	.25	−3.89	3.77	0.41	.30
		Height			0.17	0.04		<.001
		Age			0.35	0.30		.30
MLRT	1	(Constant)	.47	.22	−5.67	2.42	0.47	.02
		Height			0.15	0.02		<.001
	2	(Constant)	.49	.24	−1.94	2.96		.51
		Height			0.09	0.03	0.28	.01
		Age			0.51	0.24	2.25	.03

FRT, forward reach test; LRT, lateral reach test; MFRT, modified forward reach test; MLRT, modified lateral reach test; R, correlation; R^2^, coefficient of determination; SE, standard error; B, unstandardized regression coefficient; β, standardized coefficient.

*P* = significance level .05.

## 4 Discussion

This study aimed to establish the normative values of the PRTs (FRT, LRT, MFRT, and MLRT), investigate the association between the PRT scores and demographic/anthropometric variables, and develop the predictive equations of the PRT scores among TD Saudi children aged 6–11 years in the Eastern Province of SA.

Studies that estimated normative values among TD Saudi children in the same age range are few (13, 14). Emara et al. (13) established a normative value of the FRT for just boys in the Western Region of SA. In the current study, the boys' FRT scores were lower than those reported (13) with a difference of about 9 cm for the entire sample, yet there was a similarity of children's mean heights (both around 133 cm), weights (a 3 kg difference), and lengths of the upper limbs (a less than 1 cm difference). This discrepancy may be attributed to the length of the lower extremities, for which our participants were shorter by about 7 cm.

Tedla et al. (14) established normative values for the FRT and LRT for both sexes in the Southern Region of SA. In comparison, their FRT score was high even though the children were shorter than the children participating in the current study (10 cm difference), with no difference in weight (less than a one-kilogram difference). However, there was a similarity in the LRT score (around 13 cm). Unfortunately (14), did not examine anthropometric factors, such as UE, LE, foot length, and BOS, that could explain the differences in FRT scores.

Normative values of MFRT and MLRT were estimated by Deshmukh and Joshi (16) for Indian children aged 6–11 years. Although the Saudi children were taller and heavier than the Indian children (the differences were 6 cm and 5 kg, respectively), the mean value of the MFRT was slightly lower than that of the Indian children (the mean difference was 1.5 cm). In addition, the mean difference in the MLRT between the Indian and Saudi children was 6 cm (in favor of the Indian children). Factors that could explain the differences between the values and correlate with MLRT, such as BOS and foot, UE, and LE length, were not measured (16).

Other studies have established FRT and LRT values for Turkish (17) and Indian children (15). The procedures they used to measure FRT and LRT differed from this study (i.e., reaching with extended fingers); therefore, a comparison of the results is challenging. However, we considered that the discrepancy in the reaching distances was due to variation in their anthropometric variables (14, 17).

Consistent with previous studies, the current results revealed that the PRT scores increased with age and correlated positively with height, weight, UE length, LE length, foot length, and BOS (13–17). There was no effect of sex on the PRT scores, except for the LRT, for which boys performed better than girls, and this result was in line with (13). In that work, no significant differences were observed between boys and girls. This finding was consistent with the data reported by Butz et al. (25) in testing girls and boys aged 3–9 years. Donahoe et al. (26) reported no effect of sex on FRT values in children aged 5–15 years.

In line with Habib et al. (27), age and height were the most predictive variables for FRT. However, in the current study, age and height explained 34% of the variance in FRT, which was higher than what they found (29%). Yuksel et al. (17) reported that height was a predictive variable for FRT and LRT; they found that height and LRT explained about 68% of FRT variance, and height and FRT explained 61% of the LRT scores. In another study, Deshmukh et al. (16) correlated the scores of MFRT and MLRT and reported that they predicted each other (48%). In the current study, the LRT scores were predicted only by age.

This implies that children who are older and taller tend to exhibit better test scores compared to those who are younger and shorter. Several factors contribute to the predictive power of age. A child's balance skills can be influenced by experience and motor learning, since they gain more experience by participating in different activities and environments, whereas motor learning allows them to practice and receive feedback to improve their balance skills (28). Moreover, As individuals age, their muscle strength and joint mobility tend to increase (29), which can enhance their ability to perform reaching movements and maintain balance. Moreover, as children grow, sensory systems, such as vision and proprioception, may also improve (30). Height can influence reach test and balance as a result of its biomechanical advantages. Generally, taller individuals have longer limbs (31), which allows them to reach farther and move more freely.

This study presents four equations generated from a stepwise regression model to predict PRTs. The equations used the age and height of the children as predictive variables for FRT scores and age for LRT scores. Yuksel et al. (17) established predictive equations for FRT and LRT scores. Unfortunately, as a result of different age ranges and test procedures, we could not compare the predicted scores between our equations and theirs.

The previous literature concluded that normative values were different from population to population (13–17). This indicates that it is important to determine normative values for different populations. Our study has helped reveal that there is a diversity of test values among Saudi children in different regions (13, 14).

The study has some limitations. For example, it was performed in only one region of SA and did not include children under 6 or above 11 years of age. It is strongly recommended that different geographic regions of SA be included in further research. Other factors that play a crucial role in balance and may affect PRT results, such as trunk muscle strength and flexibility and peripheral muscle strength, in addition to the environmental factors should also be investigated (13, 14, 16).

### 4.2 Conclusion and clinical implications

The obtained normal values can be used as baseline data in the assessment of balance impairments in children aged 6–11 years in SA. Future research should evaluate and compare the scores of PRTs in different regions of SA with a larger sample size to generalize the results to the entire population of SA.

## Data Availability

The raw data supporting the conclusions of this article will be made available by the authors, without undue reservation.
